# Supplementary material for: Species Identification of Red Deer (Cervus elaphus), Roe Deer (Capreolus capreolus), and Water Deer (Hydropotes inermis) Using Capillary Electrophoresis-Based Multiplex PCR
Source: Foods. 2020 Jul 23;9(8):982. doi: 10.3390/foods9080982 (PMC7466337; doi:10.3390/foods9080982)
Supplement: Supplementary file 1 [file foods-09-00982-s001.pdf]

Supplementary Materials

Table 1. Mitochondrial gene sequences of various animals used for the sequence alignment.

Target gene	Common name	Scientific name	Accession no.	Common name	Scientific name	Accession no.
**Cyt b**	**Red deer**	***Cervus elaphus***	**JF489133.1**	Lamb	*Ovis aries*	X56284.1
		C. e. haplotype	**EU004023.1**	Goat	*Capra hircus*	MH229952.1
		C. e. xanthopygus	**AF423197**	Horse	*Equus caballus*	FJ765125.1
		C. e. songaricus	**KF781114.1**	Red kangaroo	*Macropus rufus*	KY996501.1
		C. e. elaphus	**AY044856.1**	Rabbit	*Oryctolagus cuniculus*	U07566.1
		C. e. bactrianus	**AY142327.1**	Cat	*Felis catus*	AB004238.1
		C. e. yarkandensis	**AY142326.1**	Dog	*Canis lupus familiaris*	KF926378.1
		C. e. hippelaphus	**KT290948.1**	Chicken	*Gallus gallus*	KF826490.1
		C. e. elaphus	**JX966145.1**	Duck	*Anas platyrhynchos*	MK770342.1
		C. e. maral haplotype	**KX868589.1**	Turkey	*Meleagris gallopavo*	JF275060.1
	Water deer	*Hydropotes inermis*	EU315254.1	Ostrich	*Struthio camelus*	AF338715.1
	Roe deer	*Capreolus capreolus*	KT964393.1	Goose	*Anser anser*	MN122908.1
	Beef	*Bos taurus*	AF492351.1	Pheasant	*Phasianus colchicus*	FJ752430.1
	Pork	*Sus scrofa domestica*	AP003428.1	Quail	*Coturnix japonica*	KX712089.1
**12S rRNA**	**Roe deer**	***Capreolus capreolus***	**KJ681495.1**	Red kangaroo	*Macropus rufus*	KY996501.1
		C. capreolus	**KJ681491.1**	Rabbit	*Oryctolagus cuniculus*	AJ001588.1
		C. capreolus	**JN632610**	Cat	*Felis catus*	U20753.1
		C. capreolus	**KJ681488.1**	Dog	*Canis lupus familiaris*	KF926378.1
		C. capreolus	**KJ681483.1**	Chicken	*Gallus gallus*	MH732978.1
	Red deer	*Cervus elaphus*	KP172593.1	Duck	*Anas platyrhynchos*	MH744426.1
	Water deer	*Hydropotes inermis*	EU315254.1	Turkey	*Meleagris gallopavo*	EF153719.1
	Beef	*Bos taurus*	MN714195.1	Ostrich	*Struthio camelus*	AF338715.1
	Pork	*Sus scrofa domestica*	KC469587.1	Goose	*Anser anser*	MN122908.1
	Lamb	*Ovis aries*	AF010406.1	Pheasant	*Phasianus colchicus*	FJ752430.1
	Goat	*Capra hircus*	MF573068.1	Quail	*Coturnix japonica*	KX712089.1
	Horse	*Equus caballus*	AY584828.1			
**D-loop**	**Water deer**	***Hydropotes inermis***	NC011821.1	Rabbit	*Oryctolagus cuniculus*	AJ001588.1
		H. i. argyropus	KP203884	Cat	*Felis catus*	U20753.1
		H. i. argyropus	JX254914.2	Dog	*Canis lupus familiaris*	KF926378.1
	Red deer	*Cervus elaphus*	KP172593.1	Chicken	*Gallus gallus*	KM096764.1
	Roe deer	*Capreolus capreolus*	MN485773.1	Duck	*Anas platyrhynchos*	KJ883269.1
	Beef	*Bos taurus*	AF022924.1	Turkey	*Meleagris gallopavo*	EF153719.1

Pork	*Sus scrofa domestica*	KJ789952.1	Ostrich	*Struthio camelus*	AF338715.1
Lamb	*Ovis aries*	AF010406.1	Goose	*Anser anser*	EU932689.1
Goat	*Capra hircus*	KP662715.1	Pheasant	*Phasianus colchicus*	AJ298920.1
Horse	*Equus caballus*	AY584828.1	Quail	*Coturnix japonica*	KX712089.1
Red kangaroo	*Macropus rufus*	KY996501.1			

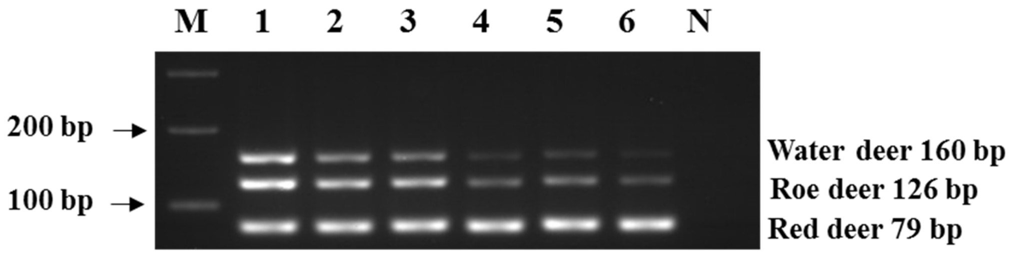

Figure 1. Detection limits of the multiplex PCR assay. Lane M: 100 bp DNA ladder, lanes 1: positive control (10 ng of each DNA from target species), lanes 2-6: 10, 5, 1, 0.5, and 0.1% roe deer and water deer in red deer, and lane N: non-template.
